# Light Converting Inorganic Phosphors for White Light-Emitting Diodes

**DOI:** 10.3390/ma3032172

**Published:** 2010-03-22

**Authors:** Lei Chen, Chun-Che Lin, Chiao-Wen Yeh, Ru-Shi Liu

**Affiliations:** 1 Department of Chemistry, National Taiwan University, Taipei 106, Taiwan; E-Mails: chichengfeiyang@yahoo.com.cn (L.C.); dovebear0310@yahoo.com.tw (C.C.L); d97223103@ntu.edu.tw (C.W.Y.); 2 School of Materials Science and Engineering, Hefei University of Technology, Hefei 230009, China

**Keywords:** light-emitting diode (LED), phosphors, luminescence efficiency, color rendering index (Ra), thermal stability

## Abstract

White light-emitting diodes (WLEDs) have matched the emission efficiency of florescent lights and will rapidly spread as light source for homes and offices in the next 5 to 10 years. WLEDs provide a light element having a semiconductor light emitting layer (blue or near-ultraviolet (nUV) LEDs) and photoluminescence phosphors. These solid-state LED lamps, rather than organic light emitting diode (OLED) or polymer light-emitting diode (PLED), have a number of advantages over conventional incandescent bulbs and halogen lamps, such as high efficiency to convert electrical energy into light, reliability and long operating lifetime. To meet with the further requirement of high color rendering index, warm light with low color temperature, high thermal stability and higher energy efficiency for WLEDs, new phosphors that can absorb excitation energy from blue or nUV LEDs and generate visible emissions efficiently are desired. The criteria of choosing the best phosphors, for blue (450−480 nm) and nUV (380−400 nm) LEDs, strongly depends on the absorption and emission of the phosphors. Moreover, the balance of light between the emission from blue-nUV LEDs and the emissions from phosphors (such as yellow from Y_3_Al_5_O_12_:Ce^3+^) is important to obtain white light with proper color rendering index and color temperature. Here, we will review the status of phosphors for LEDs and prospect the future development.

## 1. Introduction

Solid-state semiconductor lighting technology can be traced as far back as 1962 to the first semiconductor diode laser announced by Hall [1] at General Electric Research Labs in Schenectady, New York, but there was almost no application besides used in numeric displays or indicator lights in past, because the wavelengths produced by semiconductor lasers have generally been longer than 0.7 μm [1]. However, this situation was completely changed with Shuji Nakamura’s invention, who successfully fabricated double-heterostructure InGaN/GaN blue LED chips for the first time in 1993 and later in 1994 succeeded in producing l-cd-brightness high-power blue InGaN/AlGaN LEDs suitable for commercial applications [2,3,4,5,6]. Since the first commercially available white light-emitting diode (LED) was produced by Nichia Corporation in 1996, tremendous progress has been achieved in development of solid-state lighting based on InGaN semiconductors [6,7]. Besides in technique itself, a huge industrial chain has formed around white LEDs, including the upstream of the epitaxial films growth and single crystal diode chips, midstream of white LED preparation, LED packaging materials and accessories, and the downstream of the bulb-type LEDs, digital display and dot matrix display application, *etc.*

The operation of LEDs is based on spontaneous light emission in semiconductors, which is due to the radiative recombination of excess electrons and holes that are produced by the injection of current. Therefore, LEDs are not limited by the fundamental factors that still existed in conventional incandescent lamp and compact fluorescent lamp [8]. As a result, LED light sources have superior efficiency, lifetime, reliability, which makes it more energy-saving and environmental-friendly for less thermal radiation and no mercury. The organic light emitting diode (OLED) or polymer light-emitting diode(PLED) has a similar principle of operation with LED, but whose application is restricted by the effect of circumstance on organics. Here, we focus on LEDs. Currently, the application of LEDs has been extended from signal indicators initially to automobile light, traffic light, street lighting, landscape decoration, backlight of liquid crystal display (LCD) for TV sets, computers and mobile telephones, *et al.* Converting phosphors are essential components for LEDs. In this paper, we will review the technique of white light generating and the converting phosphors for LEDs to produce idea light with proper luminescence efficiency, color rendering index and thermal stability.

## 2. Principle of White Light Generating in LEDs

**Figure 1:**
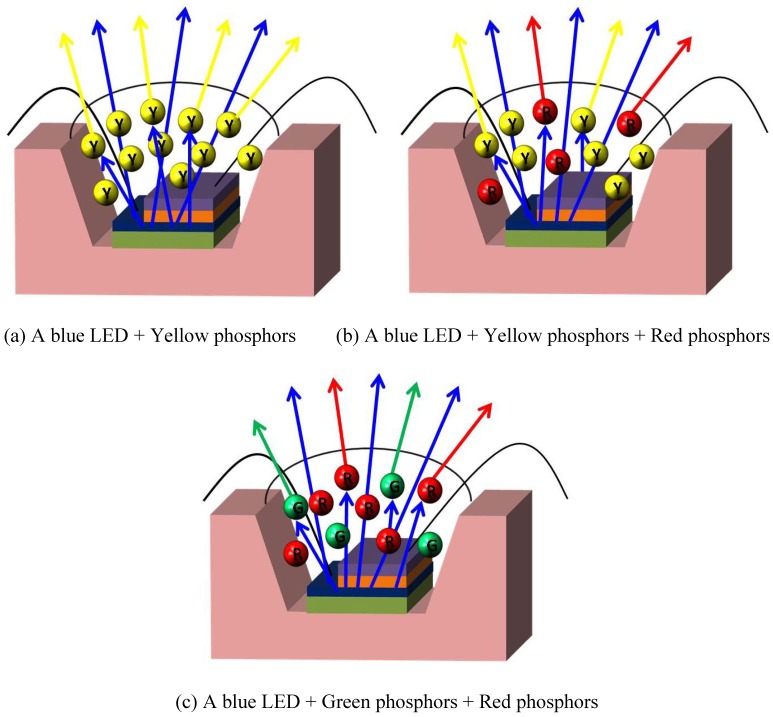
Different ways of generating white light with blue LED and phosphors.

The first commercially available white LED produced by Nichia Corporation was prepared by combining the blue emission of InGaN diode chip with the yellow luminescence from Y_3_Al_5_O_12_:Ce^3+^ (YAG:Ce^3+^) phosphor [6]. The chief drawbacks to this YAG based WLEDs are poor color rendering and seriously thermal quenching luminescence. As an alternative, combining red, green, and blue phosphors and near-ultraviolet/ultraviolet (nUV/UV) InGaN diode chips to produce white light is highly favored [9]. From the view point of industrialization, most commercially available white LEDs are prepared by pre-coating phosphors onto blue diode chips, because the luminous efficiency of blue chip based white LEDs is far higher than that of nUV/UV type. According to the phosphors adopted in LEDs, the methods of generating white light can be summarized in three different types. The first is realized by mixing blue emission from a LED diode chip and the yellow luminescence from phosphor particles, as shown in Figure 1(a), whose typical character is highly efficient but with poor color rendering index (Ra). The second is by adding proper red phosphor into the first type, as shown in Figure 1(b), through which the Ra can be improved and luminous efficiency is appropriate. The third one is prepared by combining a blue LED diode chip with red and green phosphors, as shown in Figure 1(c), whose typical character is high Ra but with low efficiency. The thermal stability of LED luminescence depends significantly on phosphors, because heat is produced continuously during LEDs operating. Presently, the phosphors that can be adopted for commercial white LEDs are summarized as following.

## 3. Converting Phosphors for LEDs

### 3.1. Yellow phosphors

#### 3.1.1. YAG:Ce^3+^

**Figure 2:**
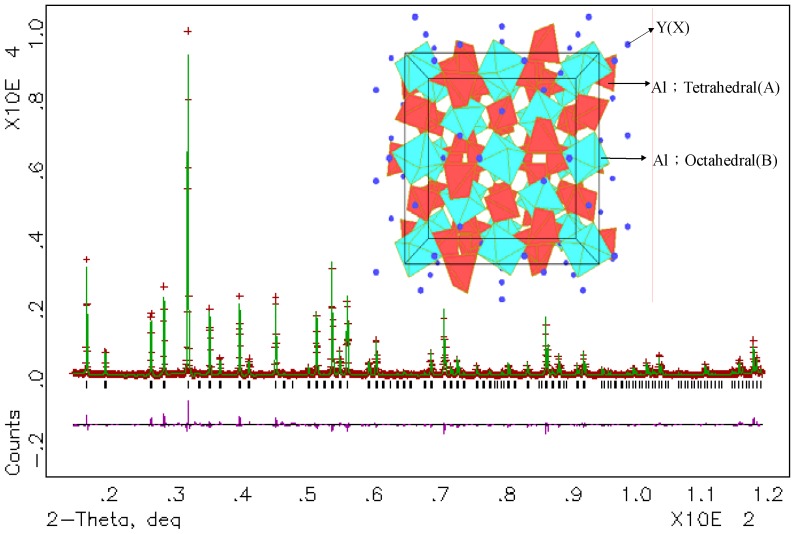
Experimental (crosses), calculated (solid line), and difference (bottom) results of XRD refinement of (Y_2.93_Ce_0.07_)Al_5_O_12_. Inset presents its three-dimensional structure.

Cerium-activated yttrium aluminum garnet (YAG:Ce^3+^) is one of most popular used phosphors to combine with single blue LEDs to generate white light. Usually, pure YAG phase is hard to achieve due to the fact that Y_2_O_3_–Al_2_O_3_ is a complex system that has two more intermediate compounds with the following composition: perovskite YAlO_3_ (YAP) and monoclinic Y_4_Al_2_O_9_ (YAM) [10,11]. With sintering temperature increasing, multiple phases transformation was experienced during Y_2_O_3_-Al_2_O_3_→YAM→YAP→YAG [11]. Synthesized with a solid-state reaction method, YAG phase can be observed at l300 °C, but the single-phase YAG can not be obtained until 1600 °C. Comparatively, single-phase YAG can be achieved at low temperature with a soft-chemical method. As a result, different synthesis methods, for example, sol-gel [12], co-deposition [13], combustion [14], and spray pyrolysis [10], were developed. Figure 2 presents experimental, calculated, and difference results of the XRD refinement of (Y_2.93_Ce_0.07_)Al_5_O_12_ at room temperature, obtained by using GSAS program [15], whose crystallographic data is shown in table 1. Y_3_Al_5_O_12_ has cubic lattice with a space group of Ia3d (230) and the lattice constants *a* = *b* = *c* = 12.0136(9) Å. The inset in Figure 2 presents the three-dimensional structure of YAG, in which Y occupies the 24(c) site with a dodecahedral (distorted cubic lattice) site and eight coordination numbers. There are two different sites for Al ions—namely, octahedral 16(a) and tetrahedral 24(d) in the lattice [16]. All of the observed peaks satisfy the reflection condition with fitted parameters as χ^2^ = 2.109, Rp = 10.02%, and Rwp = 13.69%. Therefore, Ce^3+^ ions replace the ideal sites of Y^3+^.

**Table 1:** Crystallographic Data of (Y_2.93_Ce_0.07_)Al_5_O_12_.

Atoms	x	y	z	Fraction	Uiso
Ce	0.125	0	0.25	0.023	0.0164(2)
Y	0.125	0	0.25	0.977	0.0164(2)
Al	0	0	0	1	0.0197(7)
Al	0.375	0	0.25	1	0.0171(5)
O	-0.0298(2)	0.0506(2)	0.1493(2)	1	0.0172(8)
Space group: *Ia*-3d (cubic)		Reliability factors		Interatomic distances(Å)	
Cell parameters:		R_p_ = 10.02%		Ce-O	2.300(3)
a = 12.0136(9)Å		R_wp_ = 13.69%			2.440(3)
b = 12.0136(9)Å		χ^2^ = 2.109		Al-O	1.928(3)
c = 12.0136(9)Å					1.772(2)

**Figure 3:**
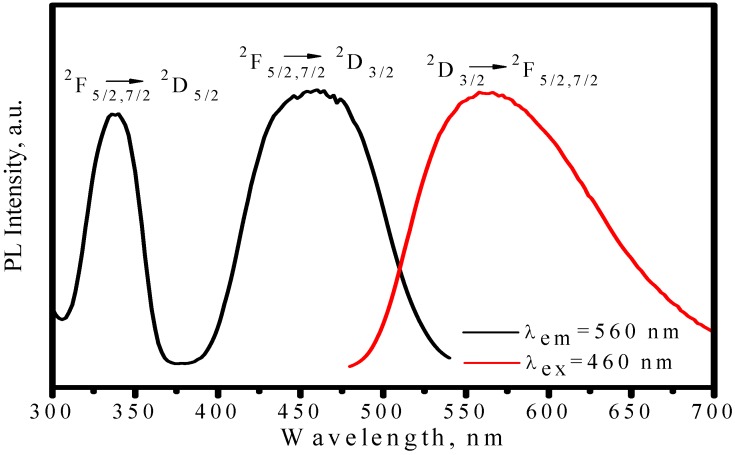
Excitation (λ_em_ = 560 nm) and emission (λ_ex_ = 460 nm) spectra of (Y_2.93_Ce_0.07_)Al_5_O_12_ phosphor.

**Figure 4:**
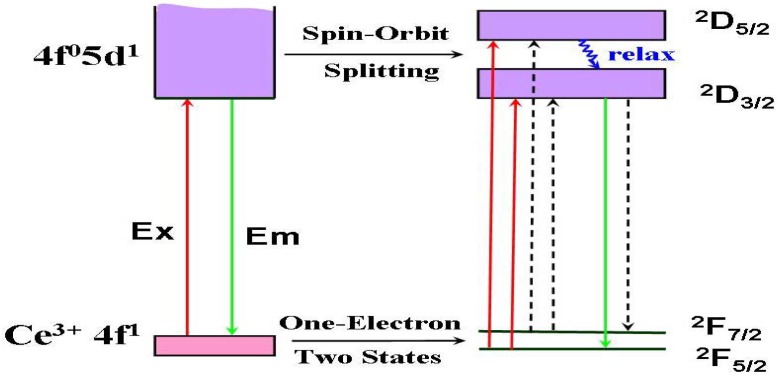
Energy-level diagram of YAG:Ce^3+^ and its excitation (EX) and emission (Em) process. The dashed lines denote the potential process.

**Figure 5:**
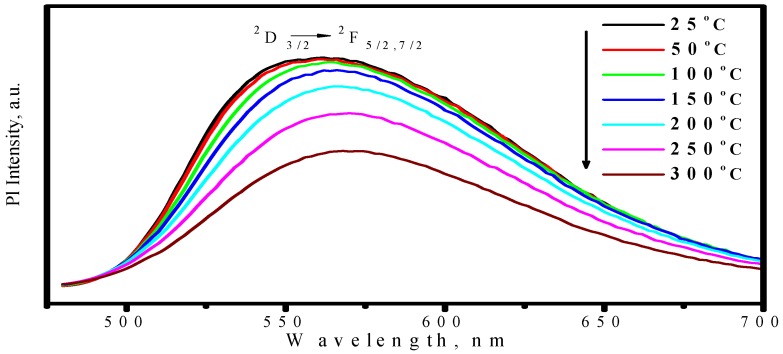
Emission spectra of YAG:Ce^3+^ upon Ce^3+^ emission at 560 nm measured at different temperature.

**Figure 6:**
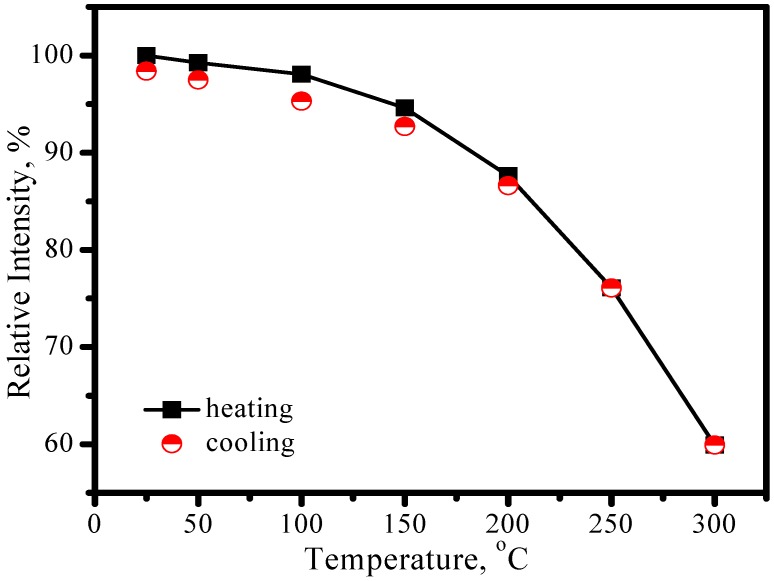
Luminescence intensity of YAG:Ce^3+^ as a function of temperature.

Figure 3 presents the photoluminescence spectra of (Y_2.93_Ce_0.07_)Al_5_O_12_, where one broad emission band with peak at nearly 560 nm is observed by exciting with 460 nm and two excitation bands with peak at about 338 and 460 nm are observed upon Ce^3+^ emission at 560 nm. As shown in Figure 4, a free Ce^3+^ ion with 4f^l^ electronic configuration has two ground states, namely ^2^F_5/2_ and ^2^F_7/2_. Once with one electron excited from 4f to 5d, the 5d electron of the exited 4f^0^5d^1^ configuration forms a ^2^D term, which is split by spin-orbit coupling and two lower energy levels of ^2^D_3/2_ and ^2^D_5/2_ states are formed [17,18]. Therefore, the excitation bands peaked at 338 and 460 nm are attributed to ^2^F_5/2_ (or ^2^F_7/2_)→^2^D_3/2_ and ^2^F_5/2_ (or ^2^F_7/2_)→^2^D_5/2_ transition, respectively. Electrons on the higher energy level of ^2^D_5/2_ state are unstable, which would relax to ^2^D_3/2_ state with electron–phonon interaction. Therefore, the emission band in Figure 3 is attributed to ^2^D_3/2_→^2^F_7/2_ or ^2^F_5/2_ transition. Because the radial wave function of the excited 5d electron extends spatially well beyond the closed 5s^2^5p^6^, its states are strongly perturbed by the crystal field. Thus, both the strongest excitation band and the strongest emission band are associated with the lowest-lying 5d state, which is affected by crystal field. According to the configuration-coordinate model, the nonradioactive transition from excited states to ground state increases with an increase of temperature. As a result, the emission peaks of YAG:Ce^3+^ redshift from 560 to 570 nm as temperature increases from 25 to 300 °C, as shown in Figure 5. Correspondingly, the luminescence intensity decreases by 6% and 41% as temperature increasing from 25 to 150 and 300 °C respectively, as displayed in Figure 6. The luminescence intensity, which is defined as the radiative energy of light source during per unit time in per unit spatial angle and is proportional to the area of emission spectrum, is obtained by integrating emission spectrum in this paper. In Figure 6, the luminescence intensity curve of heating process is almost duplicate with that of cooling process, which indicates the thermal quenching is recoverable or no thermal degradation.

#### 3.1.2. (Sr,Ba)SiO_4_:Eu^2+^

**Figure 7:**
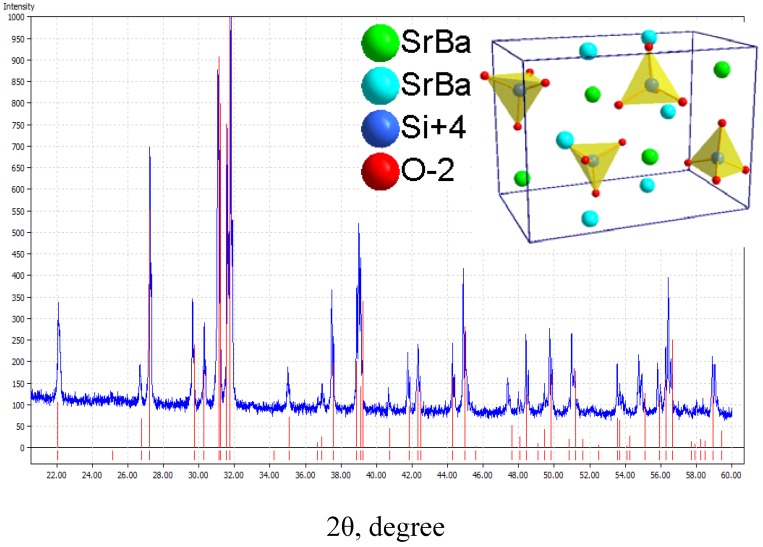
The XRD pattern of (Sr_1.7_Ba_0.2_Eu_0.1_)SiO_4_.

**Figure 8:**
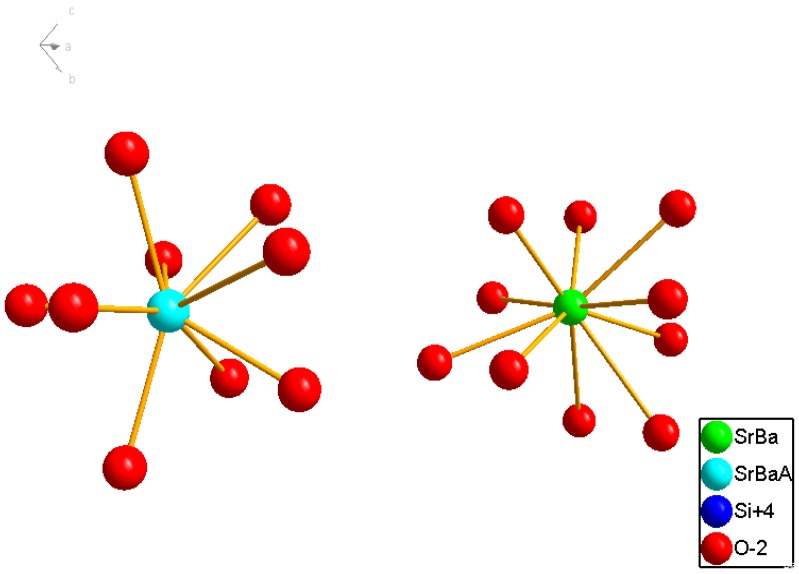
Coordination circumstance for two different Sr^2+^/Ba^2+^ site in (Sr_1.9_Ba_0.1_)SiO_4_ crystal lattice.

Besides YAG:Ce^3+^, another promising yellow phosphor for LEDs is (Sr_1.7_Ba_0.2_Eu_0.1_)SiO_4_. The XRD pattern, as shown in Figure 7, displays the same diffraction peaks as orthorhombic (Sr_1.9_Ba_0.1_)SiO_4_ (JCPDS card 76-1631). The red lines denote for the standard peaks of (Sr_1.9_Ba_0.1_)SiO_4_, and the blue line is the measured peaks. Therefore, the nominal composition of (Sr_1.7_Ba_0.2_Eu_0.1_)SiO_4_ has the same crystal structure with (Sr_1.9_Ba_0.1_)SiO_4_ [19]_._ As the inset presented in Figure 7, there two types of sites for Sr^2+^ and Ba^2+^ ions in (Sr_1.9_Ba_0.1_)SiO_4_ crystal lattice, in which Sr^2+^ and Ba^2+^ occupy the same kind of site for with the same valence and nearly radius. The coordination number of the two type of Sr^2+^ or Ba^2+^ sites are 9 and 10, as shown in Figure 8.

**Figure 9:**
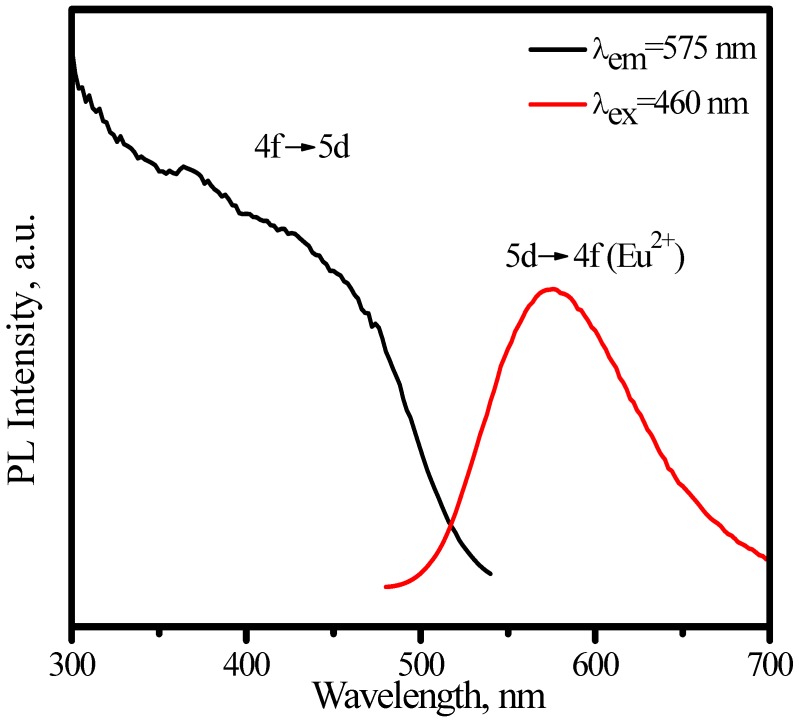
Excitation (λ_em_ = 575 nm) and emission (λ_ex_ = 460 nm) spectra of (Sr_1.7_Ba_0.2_Eu_0.1_)SiO_4_.

Figure 9 presents the photoluminescence spectra of (Sr_1.7_Ba_0.2_Eu_0.1_)SiO_4_, where one broad emission band with peak at nearly 575 nm is observed by exciting with 460 nm and one continuous band in the region of 330−500 nm is observed upon Eu^2+^ emission at 575 nm. Here, the excitation and emission are attributed to f-d transition of Eu^2+^. The 4f^7^–4f^6^5d electric dipole transition of Eu^2+^ is parity-allowed, which make it can emit efficiently. Furthermore, the emission color of Eu^2+^ varies from blue to red, strongly depending on crystal lattice circumstance, such as covalence, cation size, and the strength of the crystal field. These characters make Eu^2+^ can coordinate with LED chip to produce white light in a wide range. As the excitation spectra shown in Figure 9, (Sr_1.7_Ba_0.2_Eu_0.1_) SiO_4_ phosphor can be excited efficiently either by blue or by nUV light from LEDs.

**Figure 10:**
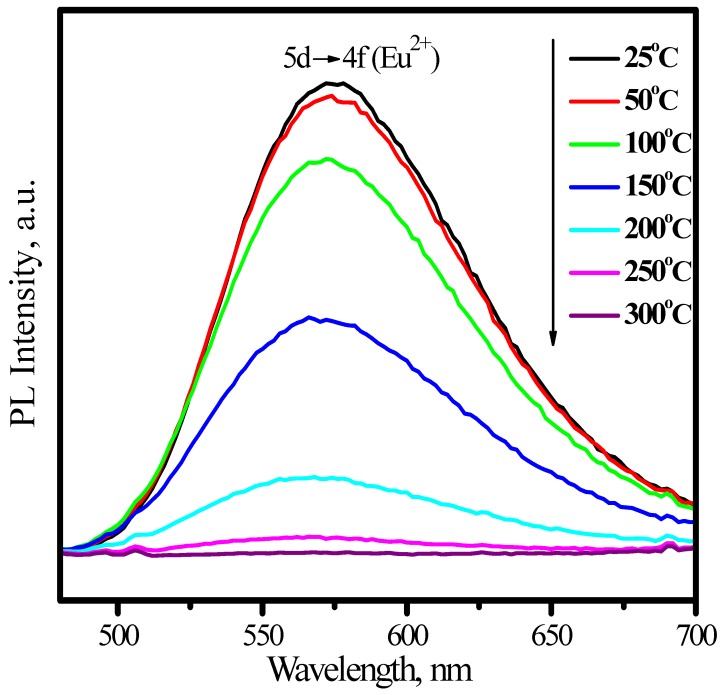
Emission spectra of (Sr_1.7_Ba_0.2_Eu_0.1_)SiO_4_ under 460 nm excitation measured at different temperature.

**Figure 11:**
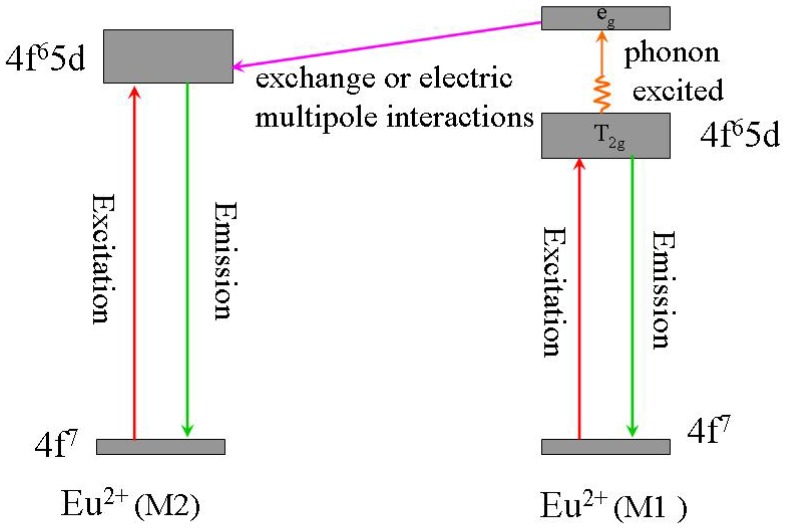
Schematic diagram of the thermal phonon-assisted tunneling of Eu^2+^ from low-energy site to high-energy site.

**Figure 12:**
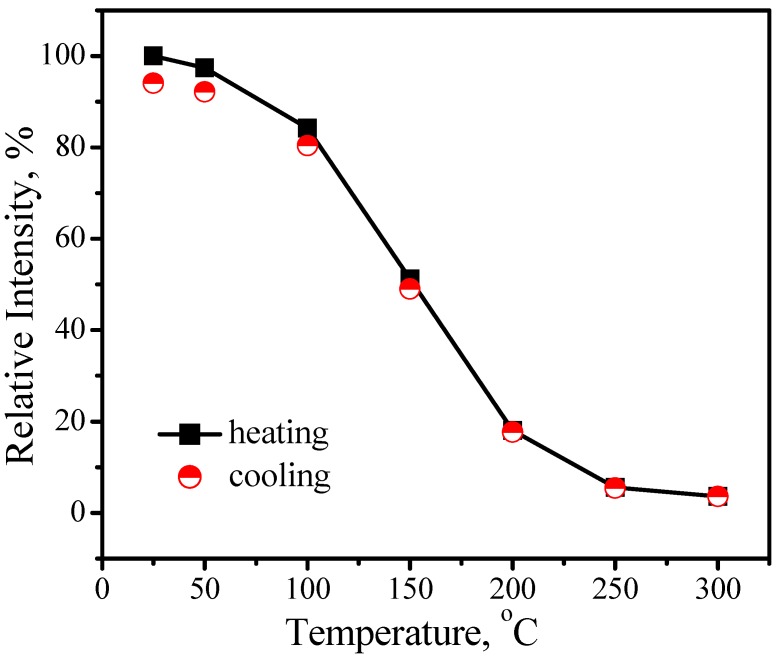
Luminescence intensity of (Sr_1.7_Ba_0.2_Eu_0.1_)SiO_4_ as a function of temperature.

Comparing with YAG:Ce^3+^, one shortcoming for (Sr_1.7_Ba_0.2_Eu_0.1_)SiO_4_ phosphor is thermal quenching. Figure 10 presents the emission spectra of (Sr_1.7_Ba_0.2_Eu_0.1_)SiO_4_ under 460 nm excitation measured at different temperature. According to configuration-coordinate model, emission spectrum usually redshifts along with temperature increasing. What is distinguished for (Sr_1.7_Ba_0.2_Eu_0.1_)SiO_4_ is that the peak of emission bands blueshift from nearly 575 to 565 nm as temperature increases from 25 to 300 °C. This is caused by the thermally active phonon-assisted tunneling from the excited states of low-energy site to the excited states of high-energy site, as shown in Figure 11. There are two kinds of site for cations in the (Sr_1.9_Ba_0.1_)SiO_4_ crystal lattice, as the inset shown in Figure 9. The doped Eu^2+^ ions usually prefer the loose environment of site, so that the emission peak of Eu^2+^ in the low concentration region locates at the higher energy side of the spectrum. As displayed in Figure 12, the luminescence intensity of the (Sr_1.7_Ba_0.2_Eu_0.1_)SiO_4_ phosphor at 150 and 300 °C are 49% and 4% of its 25 °C value, respectively. Although seriously thermal quenching is observed, the luminescence intensity curve of the heating process is almost duplicate with that of the cooling process, which indicates there is no thermal degradation or the thermal quenching is recoverable.

### 3.2. Green phosphors

#### 3.2.1. (Ba,Sr)SiO_4_:Eu^2+^

**Figure 13:**
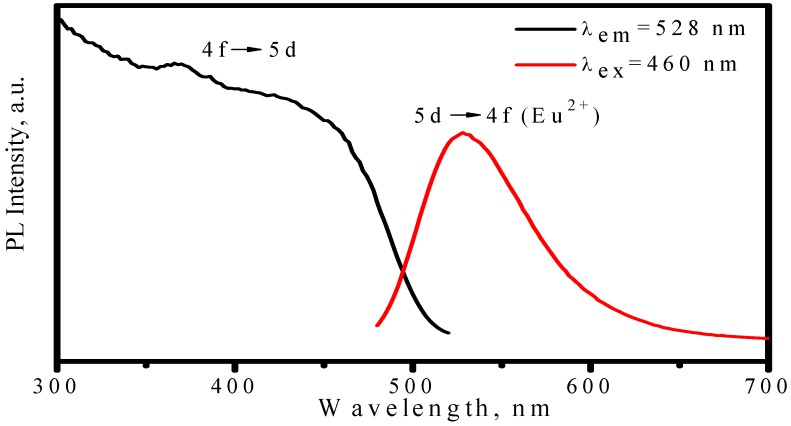
Excitation (λ_em_ = 528 nm) and emission (λ_ex_ = 460 nm) spectra of (Ba_1.1_Sr_0.7_Eu_0.2_)SiO_4_ phosphor.

**Figure 14:**
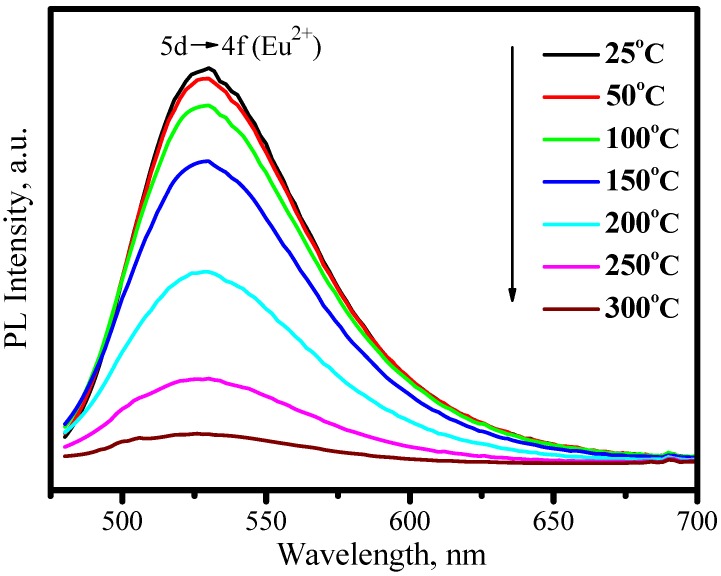
Emission spectra of (Ba_1.1_Sr_0.7_Eu_0.2_)SiO_4_ under 460 nm excitation measured at different temperature.

**Figure 15:**
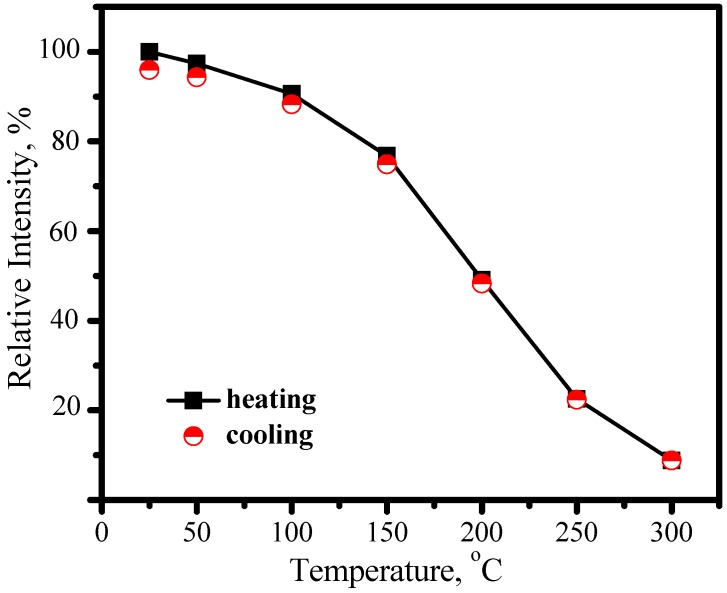
Luminescence intensity of (Ba_1.1_Sr_0.7_Eu_0.2_)SiO_4_ as a function of temperature.

As discussed above, the emission color of Eu^2+^ depends on host. As for MSiO_4_: Eu^2+^ (M = Ca, Sr, Ba) system, different emission colors can be tuned up by tailoring different cations with proper concentration, among which (Ba_1.1_Sr_0.7_Eu_0.2_)SiO_4_ is a promising green phosphor for LED. (Ba_1.1_Sr_0.7_Eu_0.2_)SiO_4_ has an isostructure with (Sr_1.7_Ba_0.2_Eu_0.1_)SiO_4_. Figure 13 presents its emission spectrum by exciting with 460 nm and excitation spectrum by monitoring at 528 nm. The strong green emission band with peak at 528 nm is assigned to 5d→4f transition of Eu^2+^. The broad continuous band in the region of 300−500 nm, which is attributed to 4f→5d transition of Eu^2+^, indicates that (Ba_1.1_Sr_0.7_Eu_0.2_)SiO_4_ phosphor can be excited efficiently either by blue or by nUV light from LEDs. The evident thermal quenching is also observed in (Ba_1.1_Sr_0.7_Eu_0.2_)SiO_4_ phosphor. As shown in Figure 14, the peak of emission bands blueshift from about 528 nm to 524 nm as temperature increases from 25 to 300 °C. Comparing with Figure 10, this blueshift of emission band is not as large as that yellow emission in (Sr_1.7_Ba_0.2_Eu_0.1_)SiO_4_, but the luminescence intensity decreases significantly with temperature increasing, as shown in Figure 15. The luminescence intensity at 150 °C and 300 °C is 77% and 9% that of at 25 °C, respectively.

#### 3.2.2. LuAG:Ce^3+^

**Figure 16:**
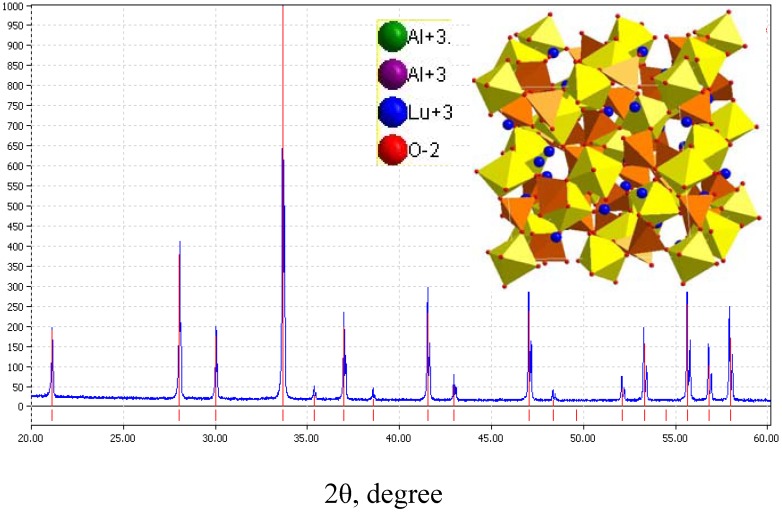
XRD pattern of (Lu_0.9_Ce_0.01_)_3_Al_5_O_13_.

The emission color of YAG:Ce^3+^ is yellow, whose properties has been discussed in above. Although Lu_3_Al_5_O_12_ (LuAG) and YAG have similar crystal structure, the emission color of Ce^3+^ in LuAG host is green, because the parity-allowed 4f ^l^-4f^0^5d^1^ transition of Ce^3+^ is also evidently affected by crystal lattice surrounding as similar to Eu^2+^. LuAG:Ce^3+^ can also be excited efficiently by the blue emission from LEDs, which make it suitable to be applied in tri-color white LEDs by adding proper red phosphor. Figure 16 presents the XRD pattern of (Lu_0.9_Ce_0.01_)_3_Al_5_O_13_, in which the red lines denote for the standard peaks of Lu_3_Al_5_O_12_ (JCPDS card 73-1368) and the blue line is the measured peaks. The inset presents its three-dimensional structure. Figure 16 shows that (Lu_0.9_Ce_0.01_)_3_Al_5_O_13_ crystallizes well as cubic with a space group of Ia3d.

**Figure 17:**
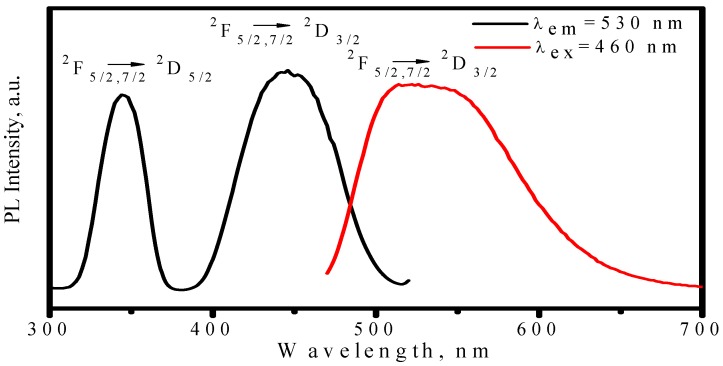
Excitation (λ_em_ = 530 nm) and emission (λ_ex_ = 460 nm) spectra of (Lu_0.9_Ce_0.01_)_3_Al_5_O_13_ phosphor.

**Figure 18:**
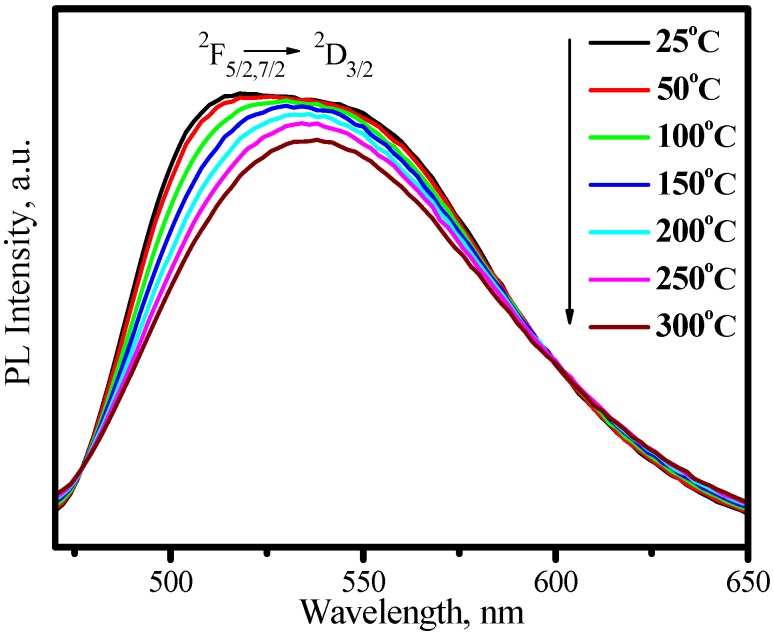
Emission spectra of (Lu_0.9_Ce_0.01_)_3_Al_5_O_13_ under 460nm excitation measured at different temperature.

**Figure 19:**
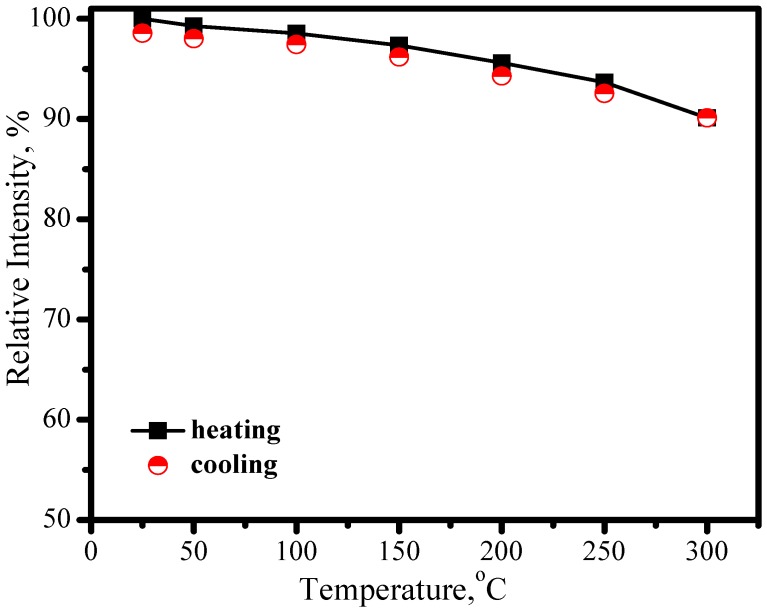
Luminescence intensity of (Lu_0.9_Ce_0.01_)_3_Al_5_O_13_ as a function of temperature.

Figure 17 presents the excitation upon Ce^3+^ emission at 530 nm and the emission spectrum under 460 nm excitation, in which two broad excitation bands in the region of 310−374 and 386−510 nm and one broad emission band in the region of 470−65 nm are observed. The redshift of the Ce^3+^ emission band with an increase of temperature was also observed in LuAG host as shown in Figure 18, which is similar to that of YAG:Ce^3+^, but the thermal stabilities of LuAG:Ce^3+^ luminescence including emission color and intensity are much better than those of YAG:Ce^3+^. As presented in Figure 19, the luminescence intensity of the LuAG:Ce^3+^ at 150 °C and 300 °C are 97% and 91% of its 25 °C value, respectively. The overlap of the luminescence intensity curves during heating and cooling process indicate that the thermal quenching is recoverable. Comparatively, the thermal stability of the green emission of LuAG:Ce^3+^ is far better than that of (Ba_1.1_Sr_0.7_Eu_0.2_)SiO_4_. The assignment of the excitation and emission spectra of LuAG :Ce^3+^ and the mechanisms of emission band redshift as well as the decrease of luminescence intensity with temperature increasing, are similar to the ones performed in YAG :Ce^3+^.

### 3.3. Red phosphors

In recent years, the research on SiN_4_-based covalent nitrides phosphor, including nitridosilicates, nitridoaluminosilicates, sialons, and nitrides et al, for LEDs has received much more attention. This is because the highly condensed SiN_4_ -based networks and the high strength of the chemical bonding between the constituent elements result in the extraordinary chemical and thermal stability of nitride materials. Furthermore, the high covalent chemical bonding in nitrides and large crystal field effect of nitrogen anion give rise to a strong nephelauxetic effect (*i.e.,* electron cloud expansion), which will reduce the energy of the excited state of the 5d electrons of the activators (e.g., Eu^2+^, Ce^3+^) and correspondingly result in larger excitation/emission wavelengths [20,21,22,23,24]. These characteristics make covalent nitrides to be ideal candidates for LED phosphors. Among all candidates for the red phosphors, M_2_Si_5_N_8_ (M = Ca, Sr, Ba):Eu^2+^ and MAlSiN_3_:Eu^2+^ (M = Ca, Sr) were demonstrated to be high promising potential phosphors for InGaN based white-light LEDs.

#### 3.3.1. (Sr,Ba)_2_Si_5_N_8_:Eu^2+^

**Figure 20:**
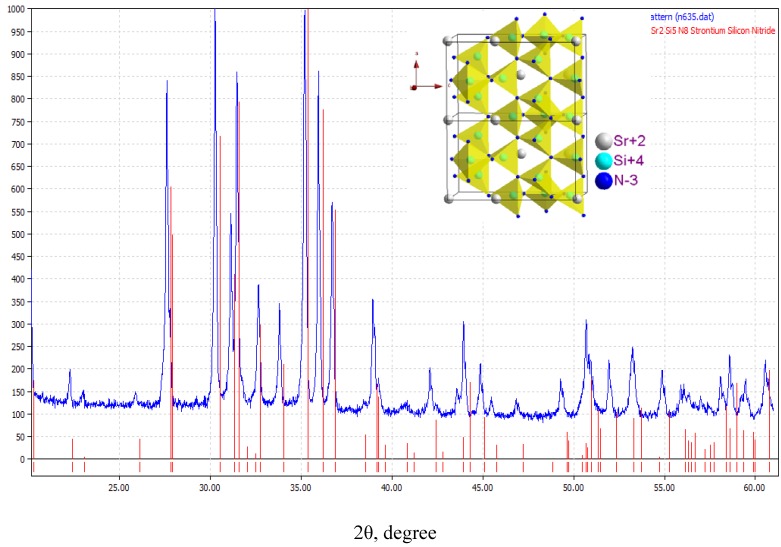
XRD pattern of (Sr_0.82_Ba_0.15_Eu_0.03_)_2_Si_5_N_8_. Inset presents its three-dimensional framework structure.

As for M_2_Si_5_N_8_:Eu^2+^ (M = Ca, Sr, Ba), they belong to different crystallographic system [25]. The isostructure of Sr_2_Si_5_N_8_ and Ba_2_Si_5_N_8_ have an orthorhombic lattice with the space group of Pmn21, however, Ca_2_Si_5_N_8_ has a monoclinic crystal system with the space group of Cc. The Eu_2_Si_5_N_8_ and M_2_Si_5_N_8_ (M = Sr, Ba) compounds are also isostructural, therefore, Eu^2+^ ions can be totally incorporated into Sr_2_Si_5_N_8_ and Ba_2_Si_5_N_8_ forming complete solid solutions. However, Eu^2+^-doped Ca_2_Si_5_N_8_ forms a limited solid solution having a monoclinic lattice. The ideal emission color can be achieved by tuning the nature of M^2+^ and their concentration. We have achieved an optimized composition as (Sr_0.82_Ba_0.15_Eu_0.03_)_2_Si_5_N_8_, whose XRD pattern is shown in Figure 20. In Figure 20, the red lines denote the standard peaks of Sr_2_Si_5_N_8_ (JCPDS: 85-0101) and the blue line is the measured data. All diffraction peaks blueshift with Ba^2+^ co-doped into Sr_2_Si_5_N_8_, because the ionic radius of Ba^2+^ is larger than that of Sr^2+^ (Ba^2+^:1.34; Sr^2+^:1.12). The (Sr_0.82_Ba_0.15_Eu_0.03_)_2_Si_5_N_8_ phosphor was synthesized with high pressure method from the precursors of EuN, Sr_3_N_2_, Ba_3_N_2_ and Si_3_N_4_ in nitrogen atmosphere in our group. The three-dimensional framework structure of Sr_2_Si_5_N_8_ is constructed by corner-sharing SiN_4_ tetrahedron, as the inset shown in Figure 20, where half the nitrogen atoms are connected to two Si neighbors (N^[2]^) and the other half have three Si neighbors (N^[3]^). There are two M sites in M_2_Si_5_N_8_ (M = Sr, Ba) crystal lattice. The N^[3]^ atoms are arranged in corrugated sheets perpendicular to [100]; and the M^2+^ ions, which are mainly coordinated by N^[2]^ atoms (E-N: 2.60−3.25 Å), are situated in channels along [100] formed by Si_6_N_6_ rings [25,26,27,28]. The coordination numbers are 8 and 10 for M_1_ and M_2_ sites, respectively [25,26,27,28]. Thus, there would be a large difference in coordination environment (and thus crystal field) for Eu^2+^ in two different M sites (the mean distance: Sr_1_–N = 2.865(6) Å *versus* Sr_2_–N = 2.928(7) Å; Ba_1_–N = 2.917(3) Å *versus* Ba_2_–N = 2.981(5) Å). However, another viewpoint considers that the coordination number are both 10, just only there are two distinct coordination surroundings for Sr^2+^, one with an average distance of 3.5 Å and the other 2.969 Å [29]. Despite of the significant controversies over this problem, two types of Sr^2+^ site are commonly accepted. As expected, two evident emission bands should be observed for the Eu^2+^ ions occupying two M sites in Sr_2_Si_5_N_8_.

**Figure 21:**
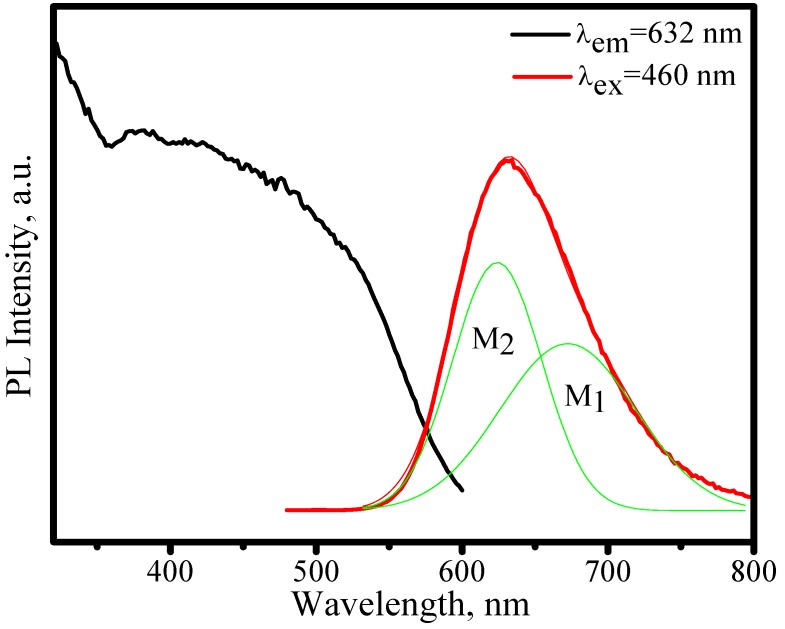
Emission (λ_ex_ = 460 nm) and excitation (λ_em_ = 632 nm) spectra of (Sr_0.82_Ba_0.15_Eu_0.03_)_2_Si_5_N_8_.

The emission (λ_ex_ = 460 nm) and excitation (λ_em_ = 632 nm) spectra of (Sr_0.82_Ba_0.15_Eu_0.03_)_2_Si_5_N_8_ are shown in Figure 21. The broad emission band peaked at 632 nm is attributed to 5d→4f transition of Eu^2+^. The strong excitation band in the region of 300−600 nm, which originates from 4f^7^→4f^6^5d^1^ transition of Eu^2+^, matches perfectly for the blue and nUV LEDs. Therefore, (Sr_0.82_Ba_0.15_Eu_0.03_)_2_Si_5_N_8_ phosphor can down-convert 380−480 nm blue-nUV light from InGaN LEDs into red emission efficiently. After Gaussian deconvolution on an energy scale, the broad emission band in Figure 20 can be well decomposed into two Gaussian components. This phenomenon is more prominent in variable temperature condition.

**Figure 22:**
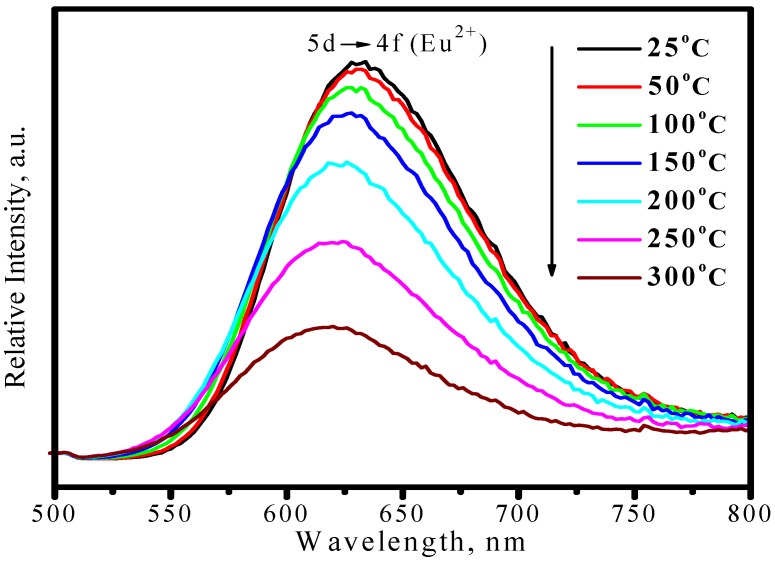
Emission spectra (λ_ex_ = 460 nm) of (Sr_0.82_Ba_0.15_Eu_0.03_)_2_Si_5_N_8_ under 460 nm excitation measured at different temperature.

**Figure 23:**
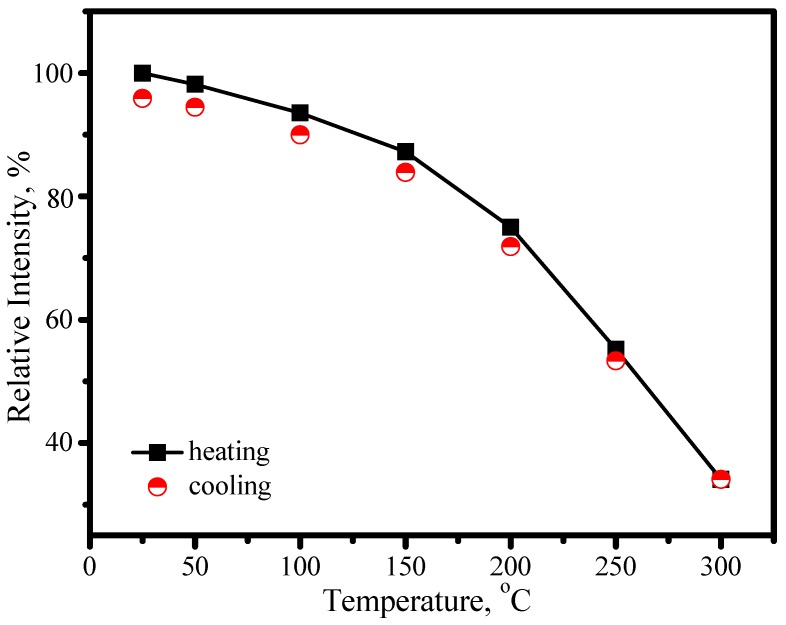
Luminescence intensity of (Sr_0.82_Ba_0.15_Eu_0.03_)_2_Si_5_N_8_ under 460 nm excitation as a function of temperature.

Figure 22 presents the emission spectra of (Sr_0.82_Ba_0.15_Eu_0.03_)_2_Si_5_N_8_ as a function of temperature, in which the emission peaks blueshit from 632 to 618 nm and the asymmetry of spectral configuration becomes more serious as temperature increases from 25 to 300 °C. Thermal quenching is also observed. As shown in Figure 23, the luminescence intensity of (Sr_0.82_Ba_0.15_Eu_0.03_)_2_Si_5_N_8_ at 150 and 300 °C are 87% and 34% of its 25 °C value, respectively

As discussed above, there are tow types site of M^2+^ in M_2_Si_5_N_8_. Eu^2+^ has the same valence and slightly smaller ionic radius as M^2+^ (Eu^2+^: 1.09, Sr^2+^: 1.12, and Ba^2+^: 1.34). Therefore, from the viewpoint of statistical thermodynamics, it is possible for Eu^2+^ ions to substitute M^2+^ sites randomly in the M_2_Si_5_N_8_ crystal lattice. It is well know that the crystal field strength, presented as D_q_, is inversely proportional to the 5th power of the bond-length R [30].

(1) $$D_{q}\propto \frac{1}{{\text{R}}^{5}}$$

A shorter bond distance implies a stronger crystal field strength. Accordingly, the decrease of the barycenter of excitation band is much higher with nephelauxetic effect and crystal field on the ^4^f^6^5d^1^→4f^7^ transition of Eu^2+^ in M_2_Si_5_N_8_. Thus, the high-energy emission, as shown in Figure 22, originates from the Eu^2+^ ions which occupy loose crystal circumstance with larger Sr-N bond length (M2); and the low-energy emission originates from the Eu^2+^ ions which occupy compact crystal circumstance with shorter Sr-N bond length (M1). Based on these, the mechanism of the blueshift of emission peaks with temperature increasing can be explained rationally according to the thermal phonon-assisted tunneling model, as shown in Figure 11.

**Figure 24:**
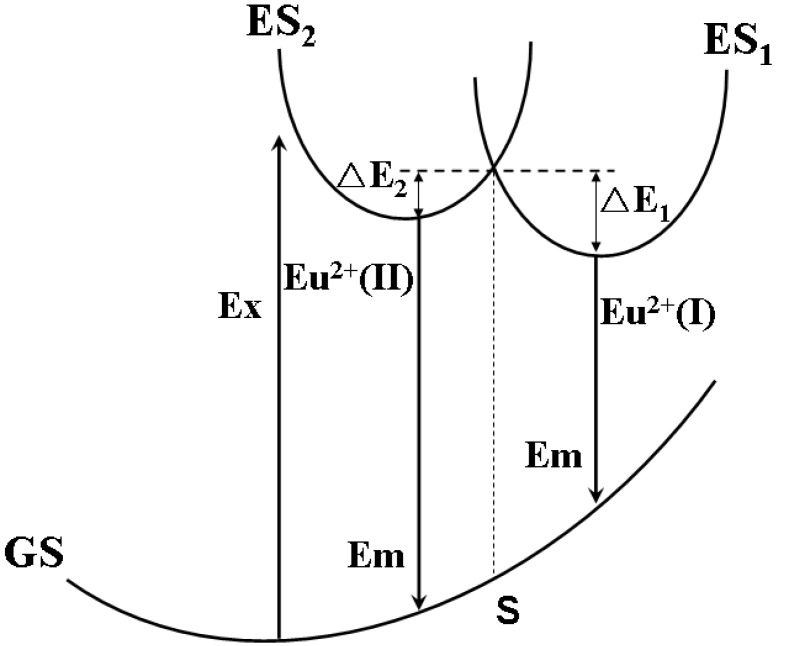
Thermally active phonon-assisted tunneling of Eu^2+^ from low-energy site to high-energy site in configuration-coordinate diagram.

Notes: GS: ground state; ES: excited state; Ex: excitation; Em: emission; S: energy barrier of excited state compared to ground state; ΔE1 and ΔE2 denote the energy barrier for the transitions of ES1→ES2 and ES2→ES1, respectively.

Besides configuration-coordinate model, there is no better physical model to elucidate thermal quenching of luminescence, which is adopted here. As revealed in Figure 24, the thermally active phonon-assisted tunneling from the excited states of low-energy (M_1_) emission band to the excited states of high-energy (M_2_) emission band in the configuration coordinate diagram increases with an increase of temperature. The energy transfer from the M_1_ site to M_2_ site through the phonon-assisted tunneling intersection of S point by overcoming the energy barrier and finally reverts to the ground state to give a shorter wavelength emission. The probability of an electron making the transition via point S is generally described as following relation:

(2) $$\alpha =s\exp(\frac{-\Delta E}{KT})$$

where K in the formula (1) presents the Boltzmann constant and s is the frequency factor. This transition is strongly dependent on the energy barrier ΔE and temperature T. The probability α generally increases with temperature increasing. Additionally, the 4f electron in the excited state crosses the intersection point between the 4f^6^5d and 4f^7^ states of Eu^2+^ ion through thermal phonon-assisted tunneling and returns to the ground state nonradiatively. Therefore, the Eu^2+^ emission peaks blueshift and luminescence intensity decreases with an increase of temperature.

#### 3.3.2. (Sr,Ca)SiAlN_3_:Eu^2+^

**Figure 25:**
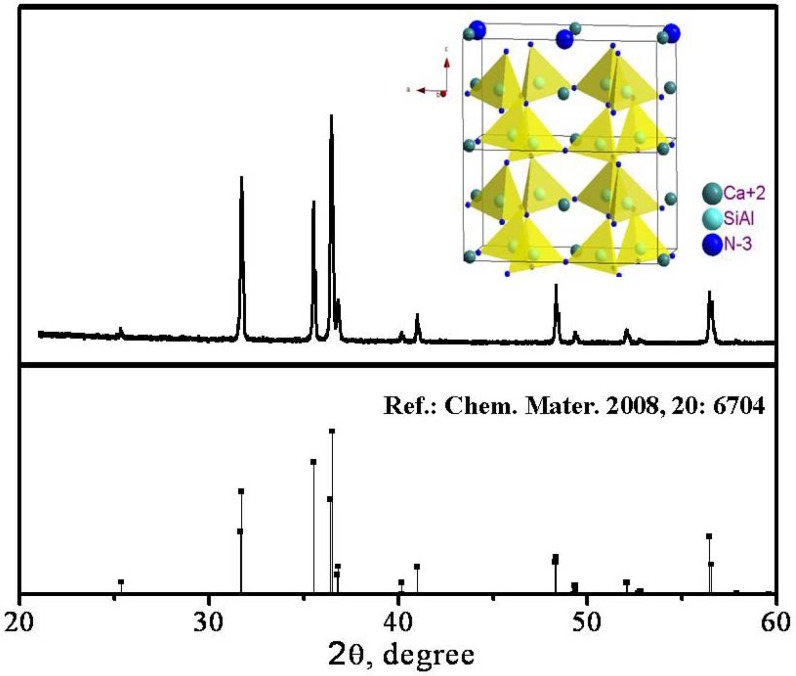
XRD pattern of (Sr,Ca)SiAlN_3_ phosphor.

CaAlSiN_3_ has an orthorhombic structure with space group of Cmc2_1_ (No. 36) [31,32]. The XRD pattern of (Sr,Ca)SiAlN_3_ phosphor synthesized in our group is consistent with reference [33], as shown in Figure 25. In accordance with the space group, Al and Si atoms in the crystal distribute randomly at 8b sites. The inset in Figure 25 presents the three-dimensional structure of CaAlSiN_3_. The tetrahedra of SiN_4_ and AlN_4_ form a six-member ring by sharing corner and Ca^2+^ ion locates in the centre of the ring. The rings consisted of the six tetrahedra form a sheet-A by linking together parallel to the a, b-plane. Another sheet B, which is equivalent to the sheet-A rotated by 21 operation, is overlaid on the sheet-A to form a rigid three dimensional framework. Two-thirds of the N atoms are coordinated with three Si atoms and one-third N atoms are coordinated with two Si atoms. Ca^2+^ ions are accommodated in cavities in the overlaid planes. The Ca site is coordinated with one N atom at a bond length of 0.2490(5) nm, two at about 0.2405(3) nm, one at about 0.2539(5) nm, and another N atom is located at 0.2586(5) nm separation. Therefore, the Ca site is four-coordinated when only the nearest-neighbor ions are taken into account, but more exactly five-coordinated. With Eu^2+^ co-doped into CaAlSiN_3_, unit cell volume is expanded linearly with Eu^2+^ concentration increasing at least up to 20 mol %, because the radius of Eu^2+^ ion is larger than that of Ca^2+^ (Ca^2+^:0.99; Eu^2+^:1.09). Therefore, Eu^2+^ ions should take place of Ca^2+^ sites in crystal lattice. The emission color and intensity of Eu^2+^ depends on Eu^2+^ concentration.

According to Uheda’s results [32], the emission intensity reaches maxima when Eu^2+^ concentration was 1.6% mol, but the wavelength of emission band with a peak at about 650 nm is too long and even some have been out of visible range. Such red emission is difficulty to mix with other emission color to form a good white light. The emission band is blueshifted by co-doping with proper Sr^2+^ and correspondingly, luminescence intensity is improved. An optimized composition, namely (Sr_0.75_Ca_0.25_)_0.98_SiAlN_3_:Eu^2+^_0.02_, has been obtained in our group.

**Figure 26:**
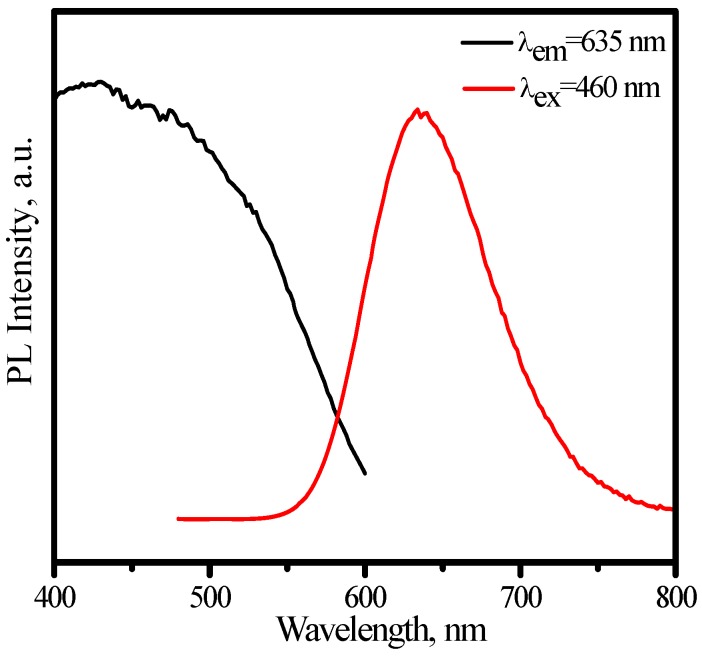
Emission (λ_ex_ = 460 nm) and excitation (λ_em_ = 635 nm) spectra of (Sr_0.75_Ca_0.25_)_0.98_SiAlN_3_:Eu^2+^_0.02_.

**Figure 27:**
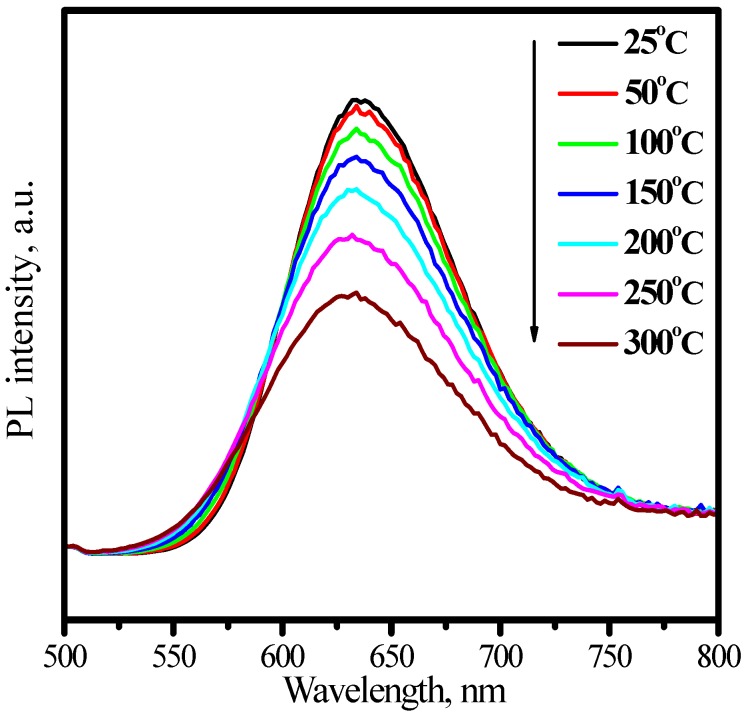
Emission (λ_ex_ = 460 nm) spectra of (Sr_0.75_Ca_0.25_)_0.98_SiAlN_3_:Eu^2+^_0.02_ under 460 nm excitation measured at different temperature.

**Figure 28:**
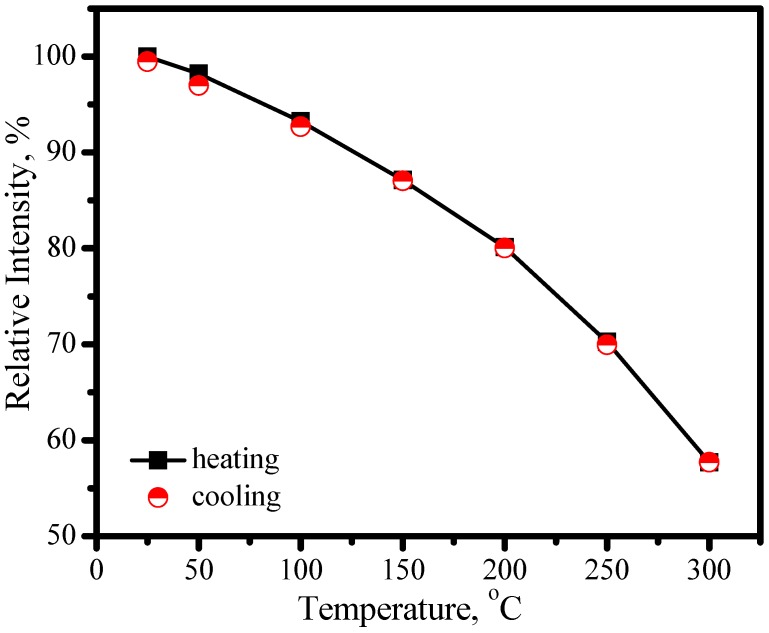
Luminescence intensity of (Sr_0.75_Ca_0.25_)_0.98_SiAlN_3_:Eu^2+^_0.02_ under 460 nm excitation as a function of temperature.

Figure 26 presents the emission spectrum under 460 nm excitation and the excitation of (Sr_0.75_Ca_0.25_)_0.98_SiAlN_3_:Eu^2+^_0.02_ by monitoring Eu^2+^ emission at 635 nm. The excitation matches perfectly for the blue and nUV LEDs, which indicates that (Sr_0.75_Ca_0.25_)_0.98_SiAlN_3_:Eu^2+^_0.02_ phosphor can down-convert 380−450 nm blue-nUV light from InGaN LEDs into red emission efficiently. The peak of emission bands blueshift from about 635 to 631 nm with an increase of temperature from 25 to 300 °C, as shown in Figure 27. The thermal quenching is also observed. The luminescence intensity at 150 and 300 °C is 87 and 58% that of at 25 °C, respectively, as shown in Figure 28. From Figure 28, we get to know that the thermal quenching is recoverable for the duplicate curve of heating and cooling process. The mechanism of the blueshift of emission bands and the thermal quenching of luminescence are similar to those of (Sr,Ba)_2_Si_5_N_8_:Eu^2+^.

## 4. Conclusions

**Figure 29:**
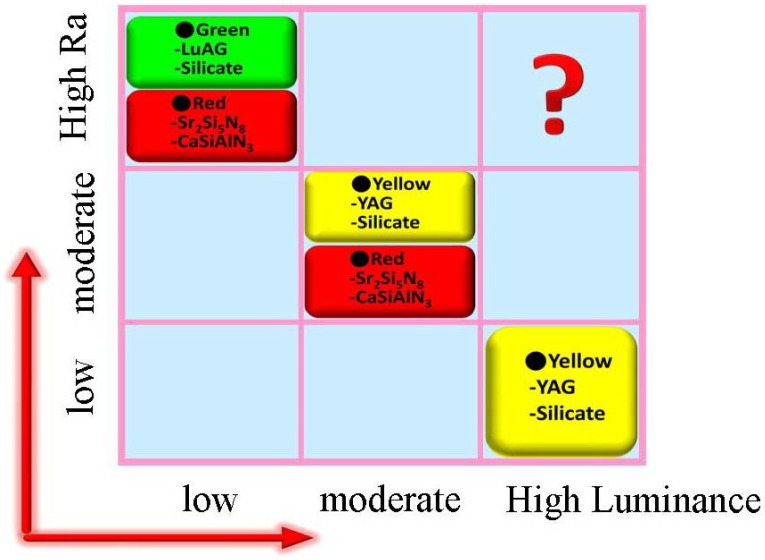
Strategies for white light generating by combining blue light-emitting diodes with different converting phosphors.

In conclusion, the strategies for generating white light by combining light-emitting diodes with converting phosphors and the phosphors that are available for producing white light with different luminescence efficiency and color rendering index properties are summarized in this paper. As shown in Figure 1 and Figure 29, to get white light with high efficiency but with low coloring index, yellow phosphors (such as YAG:Ce^3+^ or (Sr,Ba)SiO_4_:Eu^2+^) combining with blue LEDs are a good choice, which is suitable for the place that requires high brightness but without high requirement of color rendering; to obtain white light with high color rendering index but with low efficiency, green (such as LuAG:Ce^3+^ or (Ba,Sr)SiO_4_:Eu^2+^) and red phosphors (such as (Sr,Ba)_2_Si_5_N_8_:Eu^2+^ or (Sr,Ca)SiAlN_3_:Eu^2+^) combining with blue LEDs are a perfect choice, which is suitable for the place that requires high color rendering but without high requirement of efficiency; to achieve white light with appropriate color rendering index and moderate luminescence efficiency, blue LEDs and yellow phosphor (such as YAG:Ce^3+^ or (Sr,Ba)SiO_4_:Eu^2+^ ) by adding proper red phosphors (such as (Sr,Ba)_2_Si_5_N_8_:Eu^2+^ or (Sr,Ca)SiAlN_3_:Eu^2+^) are a perfect choice, which is suitable for the circumstance where both the effects of color rendering index and efficiency should be taken into account. Additionally, the white light with high color rendering index and low efficiency can also be produced by combining nUV LEDs with tri-color phosphors, but the luminescence efficiency is very lower comparing with blue LEDs. Up to now, however, the converting phosphors that are convenient to combine with blue LEDs to produce white light with high color rendering index and high luminance have not been found, as the interrogation marked in Figure 29. Therefore, the researches on LED phosphors in future will focus on this point.

## References

[1] Henini M. (2001). The blue laser diode: The complete story (2nd and extended edition): S. Nakamura, S. Pearton, G. Fasol; Springer-Verlag Berlin Heidelberg New York, 368 pages, ISBN: 3-540-66505-6. Microelectr. J..

[2] Kovac J., Peternai L., Lengyel O. (2003). Advanced light emitting diodes structures for optoelectronic applications. Thin Solid Films.

[3] Nakamura S., Senoh M., Mukai T. (1993). P-GaN/N-InGaN/N-GaN double-heterostructure blue-light-emitting diodes. Jpn. J. App. Phys..

[4] Nakamura S. (1994). InGaN-based laser diodes (In Japanese). Nikkei Electron..

[5] Nakamura S., Mukai T., Senoh M. (1994). Candela-class high-brightness InGaN/AlGaN double-heterostructure blue-light-emitting diodes. Appl. Phys. Lett..

[6] Nakamura S., Fasol G. (1997). The Blue Laser Diode: GaN Based Light Emitters and Lasers.

[7] Liu J., Lian H., Sun J., Shi C. (2005). Characterization and properties of green emitting Ca_3_SiO_4_Cl_2_:Eu^2+^ powder phosphor for white light-emitting diodes. Chem. Lett..

[8] Schubert E.F., Kim J.K. (2005). Solid-state light sources getting smart. Science.

[9] Liu R.S., Liu Y.H., Bagkar N.C., Hu S.F. (2007). Enhanced luminescence of SrSi_2_O_2_N_2_:Eu^2+^ phosphors by codoping with Ce^3+^, Mn^2+^, and Dy^3+^ ions. Appl. Phys. Lett..

[10] Mancic L., Marinkovic K., Marinkovic B.A., Dramicanin M., Milosevic O. (2010). YAG:Ce^3+^ nanostructured particles obtained via spray pyrolysis of polymeric precursor solution. J. Eur. Ceram. Soc..

[11] Li Y.X., Min Y.L., Zhou X.Z., You X.Z. (2003). Coating and stability of YAG:Ce phosphor synthesized using inorganic-organic hybrid gel method. Chin. J. Inorg. Chem..

[12] Lu C.H., Hong H.C., Jaganathan R. (2002). Sol–gel synthesis and photoluminescent properties of cerium-ion doped yttrium aluminium garnet powders. J. Mater. Chem..

[13] Zhou Y., Lin J., Yu M., Wang S.B., Zhang H.J. (2002). Synthesis-dependent luminescence properties of Y_3_Al_5_O_12_:Re^3+^ (Re = Ce, Sm, Tb) phosphors. Mater. Lett..

[14] Shi S.K., Wang J.Y. (2001). Combustion synthesis of Eu^3+^ activated Y_3_Al_5_O_12_ phosphor nanoparticles. J. Alloys Compounds.

[15] Larson A.C., Von Dreele R.B. (1994). Generalized Structure Analysis System (GSAS).

[16] Chiang C.C., Tsai M.S., Hon M.H. (2008). Luminescent properties of cerium-activated garnet series phosphor: Structure and temperature effects. J. Electrochem. Soc..

[17] Jacobs R.R., Krupke W.F., Weber M.F. (1978). Measurement of excited-state-absorption loss for Ce^3+^ in Y_3_Al_5_O_12_ and implications for tunable 5d→4f rare-earth lasers. Appl. Phys. Lett..

[18] Dong Y., Zhou G., Xu J., Zhao G., Su F., Su L., Zhang G., Zhang D., Li H., Si J. (2006). Luminescence studies of Ce:YAG using vacuum ultraviolet synchrotron radiation. Mater. Res. Bull..

[19] Catti M., Gazzoni G., Ivaldi G. (1983). Structures of twinned β-Sr_2_SiO_4_ and of α'-Sr_1.9_Ba_0.1_SiO_4_. Acta Crystallogr. C.

[20] Höppe H.A., Lutz H., Morys P., Schnick W., Seilmeier A. (2000). Luminescence in Eu^2+^-doped Ba_2_Si_5_N_8_: Fluorescence, thermoluminescence, and upconversion. J. Phys. Chem. Solids.

[21] van Krevel J.W.H., Hintzen H.T., Metselaar R., Meijerink A. (1998). Long wavelength Ce^3+^ emission in Y–Si–O–N materials. J. Alloys Compounds.

[22] van Krevel J.W.H., van Rutten J.W.T., Mandal H., Hintzen H.T., Metselaar R. (2002). Luminescence properties of terbium-, cerium-, or europium-doped *α*-sialon materials. J. Solid State Chem..

[23] Xie R.J., Mitomo M., Uheda K., Xu F.F., Akimune Y. (2002). Preparation and luminescence spectra of Calcium- and Rare-Earth (R = Eu, Tb, and Pr)-codoped α-SiAlON ceramics. J. Am. Ceram. Soc..

[24] Uheda K., Takizawa H., Endo T., Yamane H., Shimada M., Wang C.M., Mitomo M. (2000). Synthesis and luminescent property of Eu^3+^-doped LaSi_3_N_5_ phosphor. J. Lumin..

[25] Li Y.Q., van Steen J.E.J., van Krevel J.W.H., Botty G., Delsing A.C.A., DiSalvo F.J., de With G., Hintzen H.T. (2006). Luminescence properties of red-emitting M_2_Si_5_N_8_:Eu^2+^ (M = Ca, Sr, Ba) LED conversion phosphors. J. Alloys Compd..

[26] Huppertz H., Schenick W. (1997). Eu_2_Si_5_N_8_ and EuYbSi_4_N_7_. The first nitridosilicates with a divalent rare earth metal. Acta Crystallogr. C.

[27] Duan C.J., Otten W.M., Delsing A.C.A., Hintzen H.T. (2008). Preparation and photoluminescence properties of Mn^2+^-activated M_2_Si_5_N_8_ (M = Ca, Sr, Ba) phosphors. J. Solid State Chem..

[28] Piao X., Horikawa T., Hanzawa H., Machida K.I. (2006). Characterization and luminescence properties of Sr_2_Si_5_N_8_:Eu^2+^ phosphor for white light-emitting-diode illumination. Appl. Phys. Lett..

[29] Sohn K.S., Lee S., Xie R.J., Hirosaki N. (2009). Time-resolved photoluminescence analysis of two-peak emission behavior in Sr_2_Si_5_N_8_:Eu^2+^. Appl. Phys. Lett..

[30] Huang P., Cui C.E, Wang S. (2009). Synthesis and characterization of Sr_3_Al_2_O_6_:Eu^2+^, Dy^3+^ phosphors prepared by sol-gel-combustion processing. Chin. Phys. B.

[31] Uheda K., Hirosaki N., Yamamoto Y., Naito A., Nakajima T., Yamamoto H. (2006). Luminescence properties of a red phosphor, CaAlSiN_3_:Eu^2+^, for white light-emitting diodes. Electrochem. Solid-State Lett..

[32] Uheda K., Hirosaki N., Yamamoto H. (2006). Host lattice materials in the system Ca_3_N_2_–AlN–Si_3_N_4_ for white light emitting diode. Phy. Status Solidi A.

[33] Li Y.Q., Hirosaki N., Xie R.J., Takeda T., Mitomo M. (2008). Yellow-orange-emitting CaAlSiN_3_:Ce^3+^ phosphor: Structure, photoluminescence, and application in white LEDs. Chem. Mater..

